# Use of Bayesian Statistics to Reanalyze Data From the Pragmatic Randomized Optimal Platelet and Plasma Ratios Trial

**DOI:** 10.1001/jamanetworkopen.2023.0421

**Published:** 2023-02-22

**Authors:** Daniel Lammers, Joshua Richman, John B. Holcomb, Jan O. Jansen

**Affiliations:** 1 Department of Surgery, Madigan Army Medical Center and Center for Injury Science, University of Alabama at Birmingham; 2 Center for Injury Science, University of Alabama at Birmingham

## Abstract

**Importance:**

Frequentist statistical approaches are the most common strategies for clinical trial design; however, bayesian trial design may provide a more optimal study technique for trauma-related studies.

**Objective:**

To describe the outcomes of bayesian statistical approaches using data from the Pragmatic Randomized Optimal Platelet and Plasma Ratios (PROPPR) Trial.

**Design, Setting, and Participants:**

This quality improvement study performed a post hoc bayesian analysis of the PROPPR Trial using multiple hierarchical models to assess the association of resuscitation strategy with mortality. The PROPPR Trial took place at 12 US level I trauma centers from August 2012 to December 2013. A total of 680 severely injured trauma patients who were anticipated to require large volume transfusions were included in the study. Data analysis for this quality improvement study was conducted from December 2021 and June 2022.

**Interventions:**

In the PROPPR Trial, patients were randomized to receive a balanced transfusion (equal portions of plasma, platelets, and red blood cells [1:1:1]) vs a red blood cell–heavy strategy (1:1:2) during their initial resuscitation.

**Main Outcomes and Measures:**

Primary outcomes from the PROPPR trial included 24-hour and 30-day all-cause mortality using frequentist statistical methods. Bayesian methods were used to define the posterior probabilities associated with the resuscitation strategies at each of the original primary end points.

**Results:**

Overall, 680 patients (546 [80.3%] male; median [IQR] age, 34 [24-51] years, 330 [48.5%] with penetrating injury; median [IQR] Injury Severity Score, 26 [17-41]; 591 [87.0%] with severe hemorrhage) were included in the original PROPPR Trial. Between the groups, no significant differences in mortality were originally detected at 24 hours (12.7% vs 17.0%; adjusted risk ratio [RR], 0.75 [95% CI, 0.52-1.08]; *P* = .12) or 30 days (22.4% vs 26.1%; adjusted RR, 0.86 [95% CI, 0.65-1.12]; *P* = .26). Using bayesian approaches, a 1:1:1 resuscitation was found to have a 93% (Bayes factor, 13.7; RR, 0.75 [95% credible interval, 0.45-1.11]) and 87% (Bayes factor, 6.56; RR, 0.82 [95% credible interval, 0.57-1.16]) probability of being superior to a 1:1:2 resuscitation with regards to 24-hour and 30-day mortality, respectively.

**Conclusions and Relevance:**

In this quality improvement study, a post hoc bayesian analysis of the PROPPR Trial found evidence in support of mortality reduction with a balanced resuscitation strategy for patients in hemorrhagic shock. Bayesian statistical methods offer probability-based results capable of direct comparison between various interventions and should be considered for future studies assessing trauma-related outcomes.

## Key Points

**Question:**

What role could bayesian statistics play in data analysis for trauma-related clinical trials?

**Findings:**

In this quality improvement study with a post hoc bayesian analysis of the Pragmatic Randomized Optimal Platelet and Plasma Ratios Trial, there was a 93% and 87% probability that balanced transfusion strategies were superior to red blood cell–heavy approaches with regards to 24-hour and 30-day mortality, respectively.

**Meaning:**

The findings of this study suggest that bayesian approaches provide a probability-based alternative to frequentist statistics that may offer a more optimal framework for trauma-related studies.

## Teaser

This quality improvement study describes the outcomes of bayesian statistical approaches using data from the Pragmatic Randomized Optimal Platelet and Plasma Ratios (PROPPR) Trial.

## Introduction

Evidence-based medical practices remain the goal for clinical decision-making purposes in health care.^1,2^ Both clinical and basic science-focused research have greatly shaped the medical and surgical communities, resulting in a steady state of evolving practice guidelines. Prospective, randomized clinical trials (RCTs) have emerged as the criterion standard for assessing the effectiveness of new treatments in medical research due to their ability to remove biases and ensure congruent patient populations between study groups.^3^ Despite initiatives to establish the RCT as the predominate driver for clinical change, inherent difficulties with performing surgical RCTs continue to result in a high volume of observational research performed.^4,5^ These challenges are readily seen in trauma surgery, where the urgency of life-threatening scenarios exemplify the practical inabilities to recruit, consent, randomize, and allocate patients or resources to the appropriate study groups. This holds especially true during the early resuscitation phases, when it is unclear what injuries a patient may have. Furthermore, patients are frequently unable to consent for themselves and rarely have family identified during this period. For these reasons, this unique patient population differs drastically from other nontrauma, critically ill patients. Moreover, although traumatic injuries remain a commonly encountered pathology, represent a leading cause of death worldwide, and characterize a significant financial burden on the health care system, the absence of consistent funding for trauma-centered research has further resulted in a lack of high-quality RCTs compared with other fields of medicine.^6,7,8,9^

Limitations in study design and methods pose further restrictions on a study’s quality and outcomes. Most medical studies are currently structured on frequentist statistics. Frequentist approaches often focus on the concept of providing evidence against a null hypothesis to highlight significant differences between various groups through the use of *P* values. Although these approaches are the most commonly taught statistical tests in medical training, the resultant *P* values are often misinterpreted or misused by clinicians.^10^ *P* values represent the proportion of times an event as extreme, or more extreme, to the observed event will occur given that the null hypothesis is true. This definition remains conceptually complex and counterintuitive, as it centers on the assumption that there is no treatment effect present.

Bayesian statistics offer an alternative framework for data interpretation that provides the probability of a hypothesized treatment effect based on the known available data. Furthermore, bayesian approaches can preemptively incorporate prior knowledge (termed *priors*) of suspected outcomes into a study’s design. This offers the potential to provide the most accurate probabilities for each outcome in question and may strengthen a study’s findings while using fewer resources.^11,12^ As such, bayesian statistics are becoming more frequently incorporated into the medical literature, as they offer a more conceptually tangible and alternative viewpoint to frequentist statistics.

Considering these 2 statistical methods, this analysis incorporates both approaches to assess the landmark clinical trial: the Pragmatic Randomized Optimal Platelet and Plasma Ratios (PROPPR) Trial. The PROPPR Trial was the first prospective RCT to assess the resuscitation ratios of transfused units of plasma (FFP), platelets (PLT), and red blood cells (RBCs) for trauma patients in hemorrhagic shock by comparing a balanced (ie, 1 FFP:1 PLT:1 RBC; ie, 1:1:1) vs an RBC-heavy (ie, 1 FFP:1 PLT:2 RBC; ie, 1:1:2) transfusion strategy.^13^ To date, it is widely regarded as the driving force behind the current resuscitation guidelines favoring balanced transfusions in patients with traumatic injurues.^14,15,16,17^ Within this analysis we intend to provide a direct comparison between the PROPPR Trial’s published frequentist outcomes with a post hoc bayesian analysis of the trial’s data to describe the unique perspectives bayesian approaches offer to highlight the potential of incorporating these methods into future trauma-based clinical trials.

## Methods

A post hoc side-by-side analysis of the PROPPR Trial was performed between December 2021 and June 2022 to compare frequentist vs bayesian statistical techniques. Per institutional policy, this study was exempt from institutional review board approval and the requirement for informed consent due to its retrospective nature and use of publicly available, deidentified data. Manuscript preparation was guided by the Standards for Quality Improvement Reporting Excellence (SQUIRE) guidelines for reporting quality improvement studies in health care.^18^

The PROPPR Trial enrolled trauma patients between August 2012 and December 2013 who were expected to require large volume transfusions at 12 US level I trauma centers and randomized these patients to receive a 1:1:1 or 1:1:2 initial transfusion strategy to assess the effects of blood product ratios on mortality. Primary outcomes were 24-hour and 30-day mortality. The original study was designed to detect a 10–percentage point absolute difference in 24-hour mortality (11% vs 21%) and a 12–percentage point absolute difference in 30-day mortality (23% vs 35%) based on previously documented literature.^13,19^

### Statistical Analysis

Bayesian logistic regression hierarchical models were created to compare the mortality rates between resuscitation cohorts for each of the original study’s primary end points. Hierarchical models offer the ability to account for site-specific variations in practice patterns. Noninformative priors were incorporated into the models and represent a common approach that assumes a uniform distribution for all outcomes in efforts to minimize any influence on the results. The posterior probabilities and associated 95% credible intervals (CrIs) were calculated using the median 2.5th and 97.5th percentiles of the posterior distribution for each of the PROPPR Trial’s primary end points. Cohort differences within the bayesian hierarchical models were directly compared with the frequentist results originally described within the PROPPR Trial.

Additionally, posterior probabilities over a range of mortality thresholds were calculated for each of the bayesian hierarchical models to highlight the more granular possibilities offered by this approach. Each of these probabilities was characterized by an associated Bayes factor (BF) to provide an objective assessment in the form of a likelihood ratio between the competing hypotheses. The resultant BF’s degree of evidence was reported via previously accepted methods.^20^

The PROPPR Trial’s frequentist approach compared the 2 resuscitation ratios using a 2-sided Mantel-Haenszel test. The bayesian hierarchical models incorporated in this analysis used each resuscitation strategy as a fixed-level effect and each trauma center as a group-level effect. Markov chain Monte Carlo modeling via 4 chains, 1000 iterations burn-in, and 2000 saved iterations per chain with a Bernoulli family distribution was used to develop and optimize the models. All models were created using the BRMS package in R version 4.0.3 (R Project for Statistical Computing).^21,22^

## Results

The PROPPR Trial randomized 680 patients (338 in the 1:1:1 cohort vs 342 in the 1:1:2 cohort) with 546 (80.3%) male patients, a median (IQR) age of 34 (24-51) years, a median (IQR) Injury Severity Score of 26 (17-41), a total of 330 (48.5%) with penetrating injury, and a total of 591 (87.0%) with severe hemorrhage. Baseline characteristics between the 2 cohorts failed to demonstrate any differences and have been previously published elsewhere.^13^

The primary outcomes from the original PROPPR Trial frequentist analysis failed to demonstrate any significant differences in 24-hour and 30-day mortality between the 2 resuscitation strategies. At 24 hours, the 1:1:1 cohort displayed a 12.7% mortality rate compared with a 17.0% mortality rate within the 1:1:2 cohort (difference, −4.2% [95% CI, −9.6% to 1.1%]; risk ratio [RR], 0.75 [95% CI, 0.52 to 1.08]; *P* = .12), while the 30-day mortality rate was 22.4% in the 1:1:1 cohort and 26.1% in the 1:1:2 cohort (difference, −3.7% [95% CI, −10.2% to 2.7%]; RR, 0.86 [95% CI, 0.65 to 1.12]; *P* = .26). Kaplan-Meier analysis further failed to demonstrate a significant survival difference between the 2 cohorts (unadjusted log-rank test, *P* = .21).^13^

**Figure 1.**
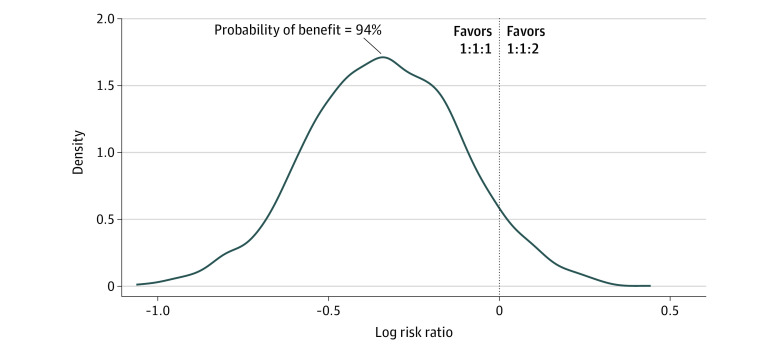
Posterior Distribution for Mortality Difference at 24 Hours

**Figure 2.**
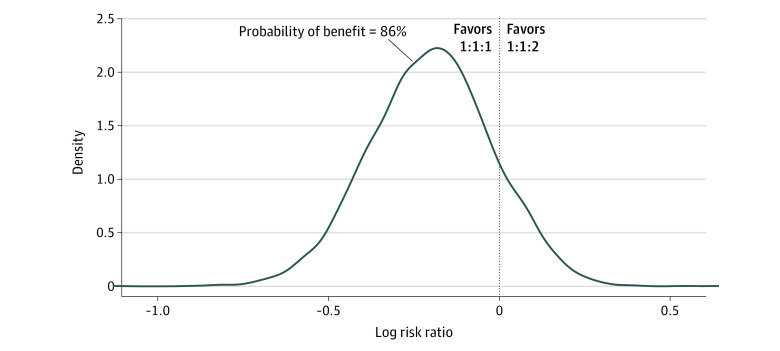
Posterior Distribution for Mortality Difference at 30 Days

Within this bayesian analysis, the probability that a 1:1:1 resuscitation was superior to a 1:1:2 strategy with regards to 24-hour mortality was found to be 93% (RR, 0.72 [95% CrI, 0.45-1.11]) (Figure 1). The associated 24-hour mortality BF was 13.7, which translates to a strong level of evidence in support of the 1:1:1 strategy.^20^ At 30 days, the models displayed an 87% probability that the 1:1:1 strategy was superior to the 1:1:2 approach (RR, 0.82 [95% CrI, 0.57-1.16]) (Figure 2). Side-by-side comparisons between statistical theories, approaches, and outcomes are displayed in Table 1. This degree of data are classified as a substantial level of evidence based on the associated 30-day BF of 6.56.^20^ Bayesian analysis for each end point over a spectrum of RR thresholds can be seen via Table 2. With an RR threshold of 0.9, there was an 85% probability that the 1:1:1 strategy was superior to the 1:1:2 strategy at 24 hours; at an RR threshold of 0.7, the probability was 46%.

**Table 1.** Comparison of Study Methods Between Frequentist and Bayesian Approaches Using the Pragmatic Randomized Optimal Platelet and Plasma Ratios Trial

Research area	Frequentist	Bayesian
Research question	Does a 1:1:1 resuscitation strategy statistically differ from a 1:1:2 strategy with regards to mortality?	What is the probability that a 1:1:1 is superior or inferior to a 1:1:2 resuscitation strategy with regards to mortality?
Assumption	Null hypothesis is true. There are no mortality differences between the 1:1:1 and 1:1:2 strategies (null hypothesis).	No preconceived assumption unless using priors. Noninformative prior results in pure assessment of data present.
Underlying theory	Assessment for statistically significant differences. Can only reject or fail to reject the null hypothesis. Cannot accept null or alternative hypothesis.	Probability based. Able to directly compare 2 hypotheses. Can accept or reject any hypothesis.
Methods	Based on *P* values and CIs. Requires power analysis. Outcomes based on series of hypothetical data sets. Secondary outcomes not powered for appropriate statistical inference. At risk for type I and type II errors.	No *P* values; uses posterior probabilities, CrIs, and Bayes factors. No power analysis required. Outcomes based on data physically present. Secondary outcomes able to be directly assessed based on findings. No risk for type I and type II errors.
Results	24-h Mortality: 12.7% (1:1:1) vs 17% (1:1:2); RR, 0.75; 95% CI, 0.52-1.08; *P* = .12; 30-d Mortality: 22.4% (1:1:1) vs 26.1% (1:1:2); RR, 0.86; 95% CI, 0.65-1.12; *P* = .26	24-hour Mortality: 93% probability that 1:1:1 is superior to 1:1:2; RR, 0.72; 95% CrI, 0.45-1.11; BF 13.7. 30-day Mortality: 87% probability that 1:1:1 is superior to 1:1:2; RR, 0.82; 95% CrI, 0.57-1.16; BF 6.56
Conclusion	The mortality rates between the 1:1:1 and 1:1:2 resuscitation strategies were not statistically different at 24 hours or 30 days. Negative trial; unable to demonstrate superiority based on frequentist approach.	There was a 93% and a 87% probability that a 1:1:1 resuscitation strategy was superior to a 1:1:2 approach at 24 hours and 30 days, respectively. Able to demonstrate superiority.

Abbreviations: CrI, credible interval; RR, risk ratio.

**Table 2.** RR Thresholds for Each Time Period Assessed^a^

Time	RR <1.0	RR <0.9	RR <0.8	RR <0.7	RR <0.6	RR <0.5
24 h						
Probability, %	93	85	68	46	22	5
Bayes factor	13.7	5.6	2.2	0.9	0.3	0.5
LOE^b^	Strong	Substantial	Anecdotal	Anecdotal	Anecdotal	Anecdotal
30 d						
Probability, %	87	71	44	19	5	0
Bayes factor	6.2	2.4	0.8	0.2	0.1	0
LOE^b^	Strong	Substantial	Anecdotal	Anecdotal	Anecdotal	Anecdotal

Abbreviations: LOE, Level of evidence; RR, risk ratio.

^a^ RR values in favor of 1:1:1 resuscitation strategy compared with 1:1:2 resuscitation strategy, with lower RR thresholds representing a stronger benefit seen within a balanced approach.

^b^ LOE based on Jeffery Scale of Evidence.^20^

## Discussion

Through failing to reject the null hypothesis, the PROPPR Trial was unable to demonstrate a significant difference in overall mortality rates between a 1:1:1 vs a 1:1:2 resuscitation strategy in patients with traumatic injuries at 24 hours and 30 days, the study’s original primary end points. Secondary outcomes suggested the 1:1:1 strategy demonstrated a reduction in death from exsanguination at 24 hours, which was not seen at 30 days.^13^ Despite being a negative study based on the primary outcomes, this trial has been largely accepted as the impetus for the wide-scale adoption of balanced resuscitation strategies in hemorrhagic shock due to the demonstrated secondary associations with decreased death from exsanguination.^14,15,16,17^ Through applying bayesian approaches to the data for the PROPPR Trial’s original primary mortality outcomes to provide an alternative evaluation metric, this analysis highlights the limitations of frequentist approaches and describes the benefits associated with bayesian strategies. In this study, we found a high probability that balanced resuscitation strategies are superior to RBC-heavy approaches with regards to mortality. Although balanced approaches are currently practiced clinically, the importance of this analysis lies within the conceptual differences between the 2 statistical approaches.

The PROPPR Trial’s frequentist results, which described no statistical difference in mortality rates between the tested strategies, fundamentally differ from our bayesian findings. The original frequentist claim is based on the resultant *P* values at each end point being greater than .05. Specifically, the *P* values for each outcome were .12 and .26 at 24 hours and 30 days, respectively.^13^ This suggests that if there truly was no difference between the 2 strategies, the probabilities of obtaining at least a 4.2% (24-hour) and 3.2% (30-day) difference in mortality would be 12% and 26%, respectively. Since these values are greater than the traditionally accepted false-positive threshold of 5%, the null hypothesis claiming no mortality difference between the cohorts could not be rejected. These data, however, do not demonstrate that the strategies are equal, which represents a commonly held misconception when analyzing negative trials. As such, the sentiment that “the absence of evidence does not provide the evidence of absence”^23^ has become popular among many statisticians. Furthermore, and an equally important concept, statistically significant results do not mathematically provide evidence of superiority. While it remains commonplace within the literature to infer superiority based on the resultant differences between cohorts, frequentist methods are unable to physically verify an alternative hypothesis (ie, a specific treatment effect exists). While the PROPPR Trial found an absence of evidence in support of the primary outcomes, had the original trial been performed using bayesian approaches, the authors likely would have come to a different primary conclusion despite using the same data.

To fully explore the meaning surrounding the differing conclusions, an understanding of the fundamental concepts and limitations inherent to frequentist statistics remains imperative. The inability to reject the null hypothesis from the original PROPPR analysis resulted in a negative study. The commonly accepted dichotomous categorization (ie, negative vs positive) of a study is a direct biproduct of the study’s *P* values. For example, the *P* value associated with the 24-hour mortality data from the PROPPR Trial was .12, which was greater than the previously set .05 threshold. Alternatively, what if the *P* value was found to be .04 (ie, a positive study)? This scenario would allow us to reject the claim that there was no treatment effect between the 2 resuscitation strategies. By dichotomizing studies based on a single *P*-value threshold, this hypothetical situation would lead to an entirely different conclusion compared with the actual *P* value of .12 at 24 hours. However, *P* value–based dichotomization represents a potentially dangerous technique, as a degree of uncertainty and error remains present in all study outcomes and can result in surprisingly varied results between identically designed trials.^24^ Understanding this concept, Gelman and Stern^25^ famously studied the associations between significant and not significant values and found that the differences between the 2 outcomes were commonly not statistically significant, highlighting the issues with arbitrary thresholds.

In addition, *P* values will inherently vary depending on the power of the study. Large sample sizes may lead to statistically significant results despite seemingly small, and even clinically inconsequential, treatment effects.^26^ Both overpowered and underpowered studies have the potential to lead to wide *P* value fluctuations.^26,27,28^ This notion has raised concerns over the practice of *P* hacking, or the process of artificially obtaining a desired value based on altering sample size and power.^29,30^ Despite this, the *P* value currently holds a tremendous amount of influence in determining the success of a trial, often without critical interpretation of the study’s data. This power and ambiguity surrounding *P* values has led to a push within the scientific community to either use a stricter threshold than .05 or abandon their use altogether.^10,31,32^

Bayesian approaches, although less well known, offer the ability to overcome the aforementioned challenges while providing more clinically useful and intuitive levels of detail compared with frequentist statistics. These methods are not reliant on *P* values to support a hypothesis and focus on providing probability estimates in direct support of a specific hypothesis.^11^ In doing so, these techniques allow the opportunity to quantify the probability of varying hypotheses based on the observed data. As demonstrated via the 93% and 87% probabilities in favor of a 1:1:1 resuscitation at 24 hours and 30 days, respectively, bayesian approaches not only allow the researcher to provide evidence in support of any hypothesis, but also provide a quantifiable degree of certainty to their level of evidence. This allows for an increased ease in interpterion for medical teams when assessing various treatment strategies. For instance, a patient may be willing to accept a 75% probability that treatment A is superior to treatment B based on their personal values and treatment goals despite treatment A not displaying a significant *P* value on a traditional frequentist analysis. This holds especially true when the treatment in question poses minimal to no risk for the patient. Moreover, quantifiable certainty can further be demonstrated through BFs, providing yet another outcome metric to directly assess the hypothesis in question.^20^

Interestingly, bayesian techniques can evaluate the probability of specific hypotheses over various threshold ranges, creating a plethora of clinically relevant data. Within our analysis, we not only evaluated the probability that a 1:1:1 resuscitation strategy was superior to a 1:1:2 approach (ie, RR, <1.0), but were also able to evaluate the degree of superiority by assigning probability values over different RR thresholds. These values can be used clinically to help further aid with the medical decision-making process. For instance, we found an 85% probability that there was at least a 10% RR reduction in mortality at 24 hours associated with the 1:1:1 approach compared with the 1:1:2 strategy (ie, RR, <0.9). However, the probability that the reduction in mortality RR using a 1:1:1 approach was greater than 30% (ie, RR, <0.7) was only 46%. From this we can deduce that the RR associated with mortality reduction using a balanced resuscitation would likely be at most between 20% and 30% based on the associated RR crossing the 50% threshold within this range. While these RR thresholds were only assessed using a uniform prior in this analysis to limit prior influence, these bayesian RR threshold analyses allow for similar techniques to be performed that incorporate a series of prior information ranging from a highly skeptical to a highly enthusiastic in support of a hypothesis in question. By using a range of informed priors, researchers can identify the ranges of potentially possible outcomes over a series of hypothetical scenarios.^33,34,35^

Finally, bayesian approaches offer the ability to create complex multivariate models in an easily digestible fashion. For instance, while a detailed analysis remains beyond the scope of this paper, one could readily assess for correlations between studied variables to elucidate novel outcome metrics, such as the probability that balanced transfusions lead to both a lower mortality and a lower number of total blood products transfused. This level of granularity provides opportunities to deepen one’s knowledge on projected treatment effects beyond the frequentist statistical methods.

### Limitations

While we believe that bayesian methods offer a more intuitive and informative analysis compared with frequentist statistical methods, we recognize that this current study is not without its limitations. This analysis was intended to provide a simple framework and example of bayesian statistics for the trauma community using a widely known clinical trial. It is not intended to provide a highly technical analysis of the trial, nor is it intended to negate the original findings of the PROPPR Trial. Because of the goals and target audience for this analysis, we chose to perform our analysis without incorporating informative prior information in efforts to mitigate complexity for the novice reader. Due to this, we chose to use a uniformed, noninformative prior in efforts to provide the simplest notion to understand. While uniform, noninformative priors offer the potential for an easily understood concept, they may not represent the most optimized statistical approach for comparing hypotheses with binary outcomes, as it is unlikely that extreme outcomes (ie, an infinitely high or low odds ratio between cohorts) are equally as probable as more realistic outcomes. Strategies that incorporate informative information ranging from skeptical to enthusiastic priors have been elegantly performed previously; however, this was not done within our current study as our intent with this analysis was to focus on theory and education as opposed to providing contrasting data to the PROPPR Trial.^35^ This notion remains especially true as balanced transfusion strategies are widely regarded as the current standard of care in trauma and, therefore, the results of this analysis should not alter clinical resuscitation practices.

## Conclusions

While both frequentist and bayesian approaches have their respective places, the benefits offered by bayesian approaches are becoming more mainstream with the widespread incorporation of advanced statistical software and improved computing power. Adoption of these techniques should be sought within future surgical trials due to the granularity of information they provide. This level of detail may ultimately provide alternative interpretations of trial outcomes and allow for improved medical decision-making. While our side-by-side comparison looking at the primary outcomes from the PROPPR Trial provides evidence in support of a mortality benefit associated with a balanced resuscitation strategy, the post hoc nature of these assumptions can only be regarded as hypothesis generating in their current form. That said, increased awareness and education on bayesian methods should be sought, and these techniques should be incorporated into future clinical trials.

## Notes

Supplement.
Data Sharing Statement
