# Supplementary material for: Use of Bayesian Statistics to Reanalyze Data From the Pragmatic Randomized Optimal Platelet and Plasma Ratios Trial
Source: JAMA Netw Open. 2023 Feb 22;6(2):e230421. doi: 10.1001/jamanetworkopen.2023.0421 (PMC9947730; doi:10.1001/jamanetworkopen.2023.0421)
Supplement: Supplement. — Data Sharing Statement [file jamanetwopen-e230421-s001.pdf]

Data Sharing Statement

Lammers. Use of Bayesian Statistics to Reanalyze Data From the Pragmatic Randomized Optimal Platelet and Plasma Ratios Trial. *JAMA Netw Open*. Published February 22, 2023. doi:10.1001/jamanetworkopen.2023.0421

Data
Data available: Yes
Data types: Deidentified participant data
How to access data: dtlammer@gmail.com
When available: With publication

Supporting Documents
Document types: None

Additional Information
Who can access the data: anyone requesting the data upon reasonable request
Types of analyses: for any purpose
Mechanisms of data availability: with a signed data access agreement
